# Real-time tracking the Li^+^-ion transition behavior and dynamics in solid Poly(vinyl alcohol)/LiClO_4_ electrolytes

**DOI:** 10.1038/srep45921

**Published:** 2017-04-05

**Authors:** Lixia Bao, Xin Zou, Xin Luo, Yanlei Pu, Jiliang Wang, Jingxin Lei

**Affiliations:** 1 School of Chemical Science and Technology, Yunnan University, Kunming 650091, P.R. China; 2 State Key Laboratory of Polymer Materials Engineering, Polymer Research Institute of Sichuan University, Chengdu 610065, P.R. China

## Abstract

To delicately track the Li-ion transport in SPEs under an external electric field (EF) is a big challenge, considering the limitation of most spectroscopic methods to monitor the real-time conformational changes and track the dynamic process. Herein, real-time Li-ion transition behavior and transport dynamics in typical poly(vinyl alcohol)/LiClO_4_ electrolytes under an external EF have been studied by combining time-resolved Fourier transform infrared (FTIR) with two-dimensional correlation FTIR spectroscopy. Results show that no migration of Li-ions has been detected when the time scale of the EF loading is at nanosecond (less than 200 *ns*). However, for the first time, Li-ions have been found to significantly transfer along the EF direction as the time scale enhances to microsecond order of magnitude and the migration period is less than 10 microseconds. The Li^+^ migration in the SPEs under an EF is a complicated process including quasi-periodic dissociation and coordination effects between Li-ion carriers and polymeric chains.

Solid polymer electrolytes (SPEs), with large potential application in rechargeable lithium batteries, solar cells, sensors and other electrochemical devices, have attracted much attention all over the world owing to the high safety and dimension stability, processibility, flexibility and design possibility^1^,^2^,^3^,^4^,^5^. However, the low ionic conductivity (σ_Li_ < 10^−4^ S/cm) of SPEs impedes their practical application and thus many efforts have been done to disclose the conductivity dependence^6^,^7^,^8^,^9^. Studies from the late 1970s to 1980s demonstrated that ionic conduction is confined to the amorphous polymer electrolytes above their glass transition temperature, *T*_*g*_, with the continuous motion of polymer chain segments^10^,^11^,^12^. Intermolecular interactions between polymeric chains and carriers are of fundamental importance for SPEs. Therefore, many studies have focused on increasing the conductivity by enhancing the content of amorphous segments (reducing *T*_*g*_ and/or crystallinity)^13^,^14^,^15^,^16^. On the contrary, Bruce has reported that the conductivity of crystalline poly(ethylene oxide)/LiXF_6_ (PEO/LiXF_6_, X = P, As, Sb) electrolytes is almost one order of magnitude higher than that of amorphous electrolytes^17^,^18^,^19^. Golodnitsky and coworkers have also qualitatively studied the impact of stretching on the conductivity of crystalline PEO/LiI electrolytes, revealing that the ionic conductivity of SPE can be effectively improved along the direction of stretching due to the formation of more ordered arrangement of molecular chains and the increasing length of PEO helixes along the longitudinal direction^20^. Recently, we have also reported that the ionic conductivity of a typical PEO_10_:LiClO_4_ electrolyte can be enhanced about one order of magnitude by the formation of more ordered crystalline structures^21^,^22^.

Despite extensive researches, the conduction mechanism of charge carriers in SPEs, especially the real-time transition behavior under an external electric field (EF), is actually unclear for the limitation of most spectroscopic methods in tracking the conformational changes of molecules or ions and monitoring the dynamic process. Furthermore, many measurements used to investigate the ion-conducting mechanism and the conductivity dependence are performed in a static environment, where the ions only randomly vibrate in a quite finite district instead of dynamically transporting along the EF direction.

From this point of view, a novel technique combining time-resolved FTIR (TR-FTIR) with two-dimensional correlation FTIR (2D-COR FTIR) that has never been used to simultaneously monitor the Li-ion transition behavior under an EF, seems to be a very promising method. For one thing, TR-FTIR technique has been a powerful tool to probe the dynamic information at sub-molecular level regarding the structure and complicated changes of molecular segments in transient processing^23^,^24^,^25^. For another, over the past two decades, generalized 2D-COR FTIR, which is generated by a cross-correlation analysis of dynamic fluctuation of IR signals induced by a certain environmental perturbations (e.g. time, stress, temperature etc.), is a well-established analytical technique to monitor the dynamic process of complex systems^26^,^27^,^28^,^29^,^30^. Herein, both the ionic transition behavior and the transport dynamics of solid poly(vinyl alcohol) (PVA)/LiClO_4_ electrolytes are extensively studied for the first time by using a combination of the TR-FTIR and 2D-COR FTIR technique under an EF, in which the time after loading the EF is considered as the perturbation (*t* = 0, nanoseconds, microseconds, and milliseconds).

## Experimental Sections

### Materials

PVA-1788 (degree of polymerization = 1700, degree of hydrolysis = 88%) was purchased from Fengchuan Chemical reagents Co., Ltd (Tianjin, China) and used as received. Analytically pure LiClO_4_ (Aldrich, USA) was dried at 105 °C under vacuum for 4 h before use. Dimethyl sulphoxide (DMSO), analytically pure, was distilled under reduced pressure and then poured into a brown reagent bottle with 5 Å sieves prior to use. All the other chemicals used were analytically pure and used as received.

### Preparation of the PVA/LiClO_4_ electrolytes

The preparation of the PVA/LiClO_4_ electrolyte membranes included the following steps. Firstly, a 5 wt.% PVA solutions were prepared by dissolving PVA powder in DMSO at 80 °C in a flask under vigorous stirring for 6 h to obtain a homogenous PVA-DMSO solution. After that, a 5 wt.% LiClO_4_-DMSO solution was quickly added into the flask under stirring when the temperature of the PVA-DMSO solution was cooled down to room temperature, and the stirring was maintained for 24 h at room temperature to form the setting SPE solutions with the hydroxyl (HO)/Li ratio of 10/1, 8/1, and 6/1, respectively. Then, the resulting homogenous solutions were poured into a special poly (tetrafluoroethylene) (PTFE) mold and dried at 120 °C under vacuum for 48 h to form a thin SPE membrane (ca. 150 μm). For simplicity, the obtained membranes were named as SPE-10, SPE-8, and SPE-6 for the HO/Li ratio of 10/1, 8/1, and 6/1, respectively. Finally, the SPE membranes with the size of 40 mm × 10 mm × 0.15 mm were further assembled between two bronze electrodes of a specially designed chamber in an argon-filled glove box.

### TR-FTIR combined with 2D-COR FTIR measurements

Time-resolved *in situ* infrared analysis of the SPE membranes was performed using a Thermo-8700 spectrometer equipped with a specially designed argon-filled specimen chamber in the wavenumber range of 2900–3500 cm^−1^ and with a resolution of 4 cm^−1^ (Figure S1). A MCT detector cooled with liquid nitrogen was used. Step scan test (SST) measurements were carried out with the time interval of 10 ns, 1 μs, and 1 ms, respectively. A DC voltage of 4 V (approaching to the real working voltage of most Li-ion batteries) was simultaneously applied to the SPE membranes. The resulting series (21) spectra were further computed by using a 2D-IR analytical software (2DCS, version 3.0, designed and developed by Prof. Zhou Tao in our institute) with the average spectrum as reference spectrum to obtain corresponding general 2D-COR and perturbation-correlation moving-window (PC-MW) 2D-COR contour maps.

## Results and Discussion

### TR-FTIR and 2D-COR FTIR results

**Figure 1:**
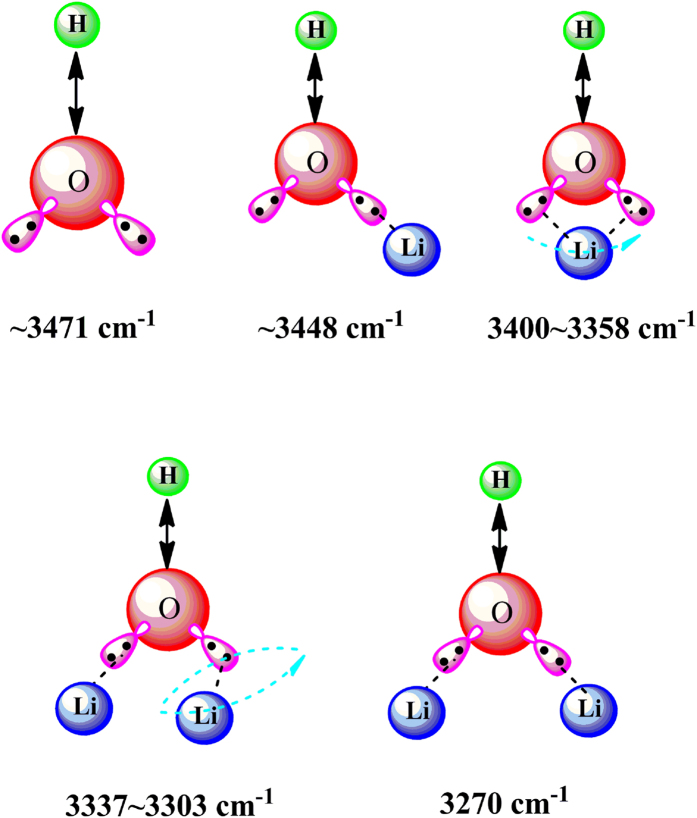
Potential coordination forms between hydroxyl groups of PVA macromolecules and Li-ions.

Characteristic peaks in the wavenumber range of 3250–3500 cm^−1^ stand for hydroxyl groups with different intermolecular interactions (i.e. hydrogen bonds). However, only one wide characteristic band in the wavenumber range of 3250–3500 cm^−1^ is usually observed when a typical 1D FTIR technique is utilized under a static circumstance and thus much useful dynamic information will be missed, while now we can divide the overlapped wide characteristic band in the wavenumber range of 3250–3500 cm^−1^ into different isolated hydroxyl groups under an external EF. Characteristic peaks at around 3500 and 3250 cm^−1^ are derived from loose hydroxyl groups and close hydroxyl groups, respectively. Potential hydroxyl groups chemically bonded on the PVA macromolecules under different micro-environments have been schematically shown in Fig. 1. Consequently, dynamic interactions between the hydroxyl groups and Li-ions in this article, stronger than that between neighboring hydroxyl groups, can be indirectly revealed by analyzing the real-time variations of both the position and the number of hydroxyl groups in the wavenumber range of 3250–3500 cm^−1^. Accordingly, real-time dynamic transition behavior of Li-ions under an EF can be also monitored by tracking the variation of corresponding characteristic peaks.

**Figure 2:**
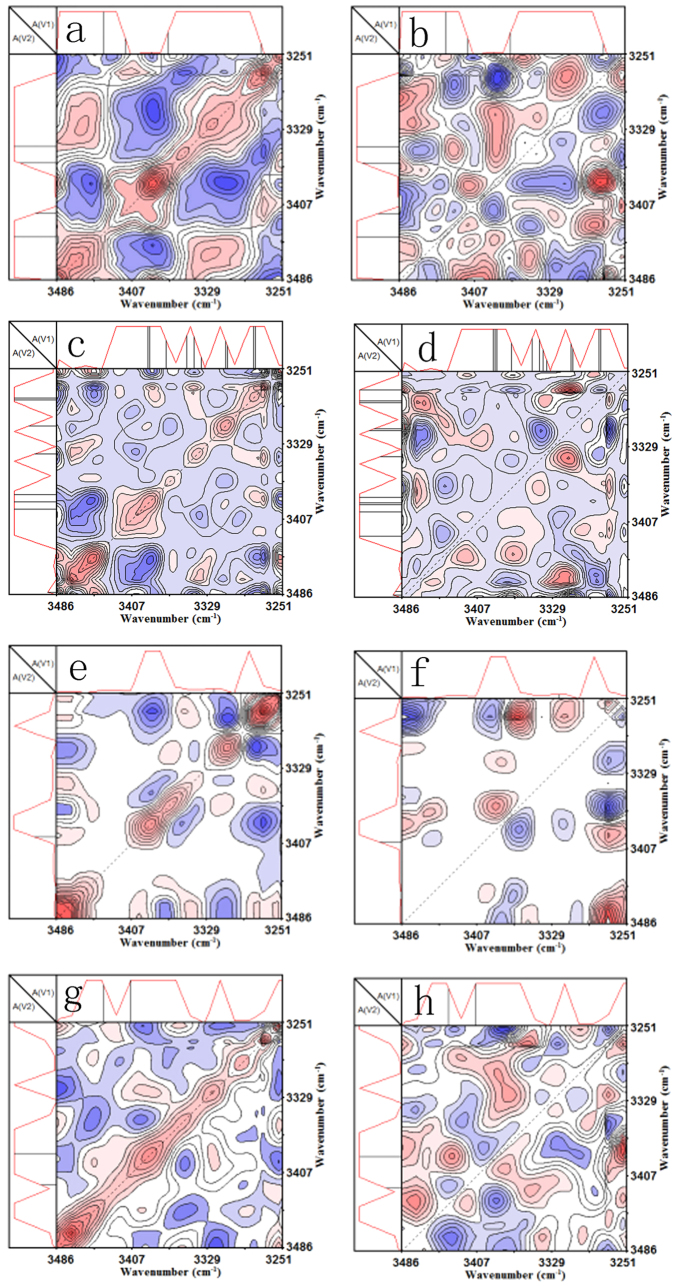
2D-COR FTIR contour maps of the SPE-10 in the wavenumber range of 3250–3500 cm^−1^ without EF (**a,b**) and with the external voltage of 4 V (**c**–**h**), (**a,c,e,g**) synchronous spectra and (**b,d,f,h**) asynchronous spectra.

Figure 2a and b show the influence of time scale (0–200 *ns*) on the synchronous and asynchronous contour map of the SPE-10 in the wavenumber range of 3250–3500 cm^−1^ without an external EF (i.g. 0 V), respectively. The 1D reference spectra (average spectrum) of SPE-10 electrolyte at the same range have been also presented at the top and the side of contour maps. In Fig. 2a, five auto-peaks at around 3448, 3400, 3337, 3303, and 3270 cm^−1^ are detected on the diagonal, and one positive and three negative cross-peaks at about Φ_1_(3448, 3270) and Φ_2_(3400, 3337), Φ_3_(3368, 3270), Φ_4_(3303 cm^−1^, 3270 cm^−1^) are observed in the off-diagonal area. In the case of Fig. 2b, two positive and four negative cross-peaks at about Ψ_1_(3448, 3303), Ψ_2_(3386, 3270) and Ψ_3_(3471, 3448), Ψ_4_(3448, 3386), Ψ_5_(3400, 3303), Ψ_6_(3303 cm^−1^, 3270 cm^−1^) have been found in the off-diagonal area. Consequently, the single wide peak with the wavenumber range of 3250–3500 cm^−1^ can be exactly separated into eight isolated hydroxyl groups (i.e. 3471, 3448, 3400, 3386, 3368, 3337, 3303, 3270 cm^−1^) even though the EF is not loaded between the specimen. The splitting of the typical single peak is probably attributed to both the microcosmic thermal vibrations of hydroxyl groups and the high sensitivity of the used 2D-COR FTIR technique, which is exactly the advantage of 2D-COR FTIR. Moreover, the splitting of the single peak implies that there are probably many Li-ion forms in the SPE-10 instead of one unique coordinated state when the external EF is not loaded, originating from that the LiClO_4_ salts have been wholly dissolved (coordinated) into the SPE-10 host. To further evaluate the transition of Li-ions under an EF, a voltage of 4 V has been loaded on the SPE specimen with different time interval (10 *ns*, 1 *μs*, and 1 *ms*), and the relevant results are presented in Fig. 2c–h. Figure 2c and d clearly display the effect of time scale (0–200 *ns*) on the synchronous and asynchronous contour map in the wavenumber range of 3250–3500 cm^−1^ with the EF of 4 V, respectively. Seven auto-peaks at around 3471, 3448, 3386, 3368, 3337, 3303, and 3270 cm^−1^ are observed on the diagonal in Fig. 2c. Furthermore, three positive and five negative cross-peaks at about Φ_1_(3471, 3337), Φ_2_(3337, 3270), Φ_3_(3386, 3270) and Φ_4_(3448, 3386), Φ_5_(3448, 3270), Φ_6_(3368, 3303), Φ_7_(3400, 3337), Φ_8_(3400 cm^−1^, 3303 cm^−1^) are observed in the off-diagonal area. In Fig. 2d, three positive and four negative cross-peaks at about Ψ_1_(3471, 3368), Ψ_2_(3448, 3303), Ψ_3_(3303, 3270) and Ψ_4_(3448, 3400), Ψ_5_(3440, 3368), Ψ_6_(3471, 3303), Ψ_7_(3337 cm^−1^, 3303 cm^−1^) have been found in the off-diagonal area. Correspondingly, the wide peak with the wavenumber range of 3250–3500 cm^−1^ can be divided into eight isolated hydroxyl groups (i.e. 3471, 3448, 3400, 3386, 3368, 3337, 3303, 3270 cm^−1^). Remarkably, both the number and the position of characteristic peaks are almost unchanged despite the loading of the EV. Li-ions in the SPE-10 are probably still in a static case or only randomly vibrate in a very limited district under the external EF when the loading time scale ranges from 0 to 200 ns, suggesting that there is not enough time for the coordinated Li-ions to effectively disassociated from the ligands (i.e. lone-pairs of the hydroxyl groups).

Both the synchronous and the asynchronous contour maps in the wavenumber range of 3250–3500 cm^−1^ of the SPE-10 are found to be significantly changed with increasing the time scale to microsecond order of magnitude (see Fig. 2e and f). In Figure 2e, five auto-peaks at around 3471, 3400, 3358, 3303, and 3270 cm^−1^ are observed on the diagonal. Only one positive and one negative cross-peaks at about Φ_1_(3358, 3270) and Φ_2_(3400 cm^−1^, 3270 cm^−1^) are detected in the off-diagonal area. Two positive and three negative cross-peaks at about Ψ_1_(3400, 3337), Ψ_2_(3303, 3270) and Ψ_3_(3471, 3303), Ψ_4_(3400, 3270), Ψ_5_(3358 cm^−1^, 3270 cm^−1^) have been detected in the off-diagonal area in Fig. 2f. Totally, the single wide peak at the wavenumber range of 3250–3500 cm^−1^ can be further divided into six isolated hydroxyl groups when the time scale increases to microseconds, including characteristic peaks of 3471, 3400, 3358, 3337, 3303, 3270 cm^−1^. Comparing the number and the position of corresponding characteristic peaks in Fig. 2e and f with those in Fig. 2a to d, it can be found that characteristic peaks at around 3448 and 3386 cm^−1^ have disappeared. And the peak at 3368 cm^−1^ has been accordingly changed into 3358 cm^−1^, showing about a 10 cm^−1^ blue-shift of the characteristic peak. Therefore, the result in Fig. 2e and f indirectly demonstrates that the form of Li-ions has apparently changed when the loading time (perturbation) under the external EF increases to microsecond (less than 20 *μs*) order of magnitude. The significant change of characteristic peaks probably implies that the coordinated Li-ions have migrated along the direction of potential reduction once the loading time achieves microsecond order of magnitude.

In Fig. 2g, five auto-peaks at around 3471, 3386, 3358, 3303, and 3270 cm^−1^ are detected on the diagonal, and only two negative cross-peaks at about Φ_1_(3448, 3358) and Φ_2_(3400 cm^−1^, 3358 cm^−1^) are observed in the off-diagonal area. In Fig. 2h, five positive and two negative cross-peaks at about Ψ_1_(3471, 3448), Ψ_2_(3448, 3386), Ψ_3_(3400, 3303), Ψ_4_(3358, 3303), Ψ_5_(3386, 3303), and Ψ_6_(3471, 3337), Ψ_7_(3400 cm^−1^, 3378 cm^−1^) have been observed in the off-diagonal area. Similarly, the single wide peak with the wavenumber range of 3250–3500 cm^−1^ can be also separated into eight isolated hydroxyl groups (i.e. 3471, 3448, 3400, 3386, 3358, 3337, 3303, 3270 cm^−1^) as the loading time of the EF has reached up to millisecond order of magnitude. Moreover, the characteristic peaks at the time scale of millisecond are very different from those at the time scale of microsecond. Specifically, the disappeared characteristic peaks at 3448 and 3386 cm^−1^ at the microsecond scale have appeared again at the millisecond and the peak at 3358 cm^−1^ is also detected, revealing that there is probably a periodic change of the hydroxyl groups with increasing the loading time of the external EF, that is, Li-ions in the SPE-10 are apt to periodically transfer along the direction of the external EF once the loading time surpasses a certain critical value (achieving microsecond order of magnitude). More details are listed in Table 1. The 2D-COR FTIR contour maps and the detailed data of characteristic peaks of SPE-8 and SPE-6 are shown in Figure S2 and Table S1, and Figure S3 and Table S2, respectively.

According to the well-known *Noda* rules, asynchronous 2D correlation spectra consisting of only cross peaks are useful to interpret the kinetics of the chemical/physical reactions and the actual sequence of individual reaction processes, then the appearance sequence of characteristic peaks under a certain perturbation (i.g. which characteristic peak of hydroxyl groups will first response to the external stimulus, and which one will be the next) can be easily obtained by simultaneously analyzing the value of both Ψ_i_(*X, Y*) and Φ_i_(*X, Y*) × Ψ_i_(*X, Y*)^27^,^28^,^29^. And the result regarding the variation sequence of the mentioned -OH groups under the EF when the loading time scale is at the range of 1–20 *μs* is listed as follows.


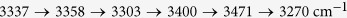


**Figure 3:**
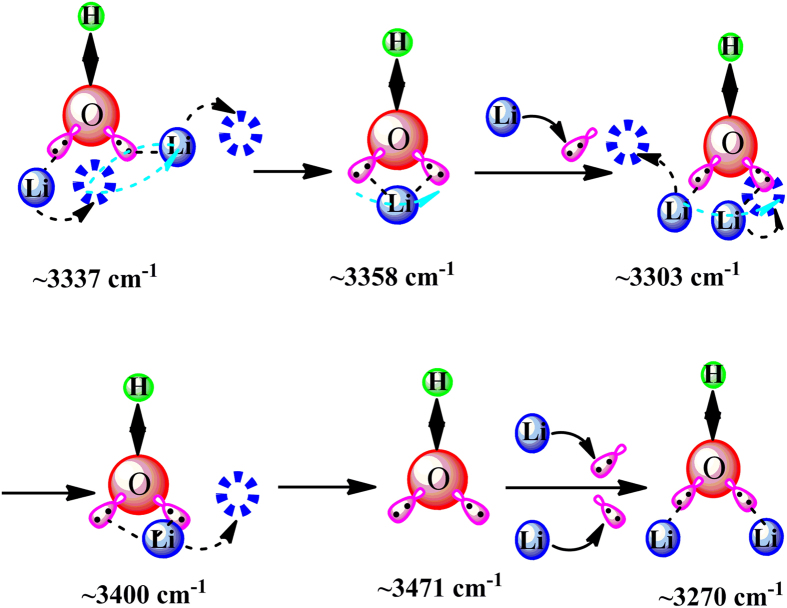
Schematically microcosmic transition mechanism of Li-ions in the SPE-10 under the EF of 4 V with the loading time scale of 1–20 *μs*.

The above variation sequence further reveals that the bonded hydroxyl groups will first change from a relatively close form (3337) into a loose one (3358 cm^−1^) and then sequentially into other forms (3303, 3400, 3471, and 3270 cm^−1^) under the EF as the loading time is up to microsecond order of magnitude, indicating the periodic transition of Li-ions under the EF. The migration process of Li-ions of the SPE-10 under the EF with loading time scale of 1–20 *μs* has been schematically shown in Fig. 3.

**Figure 4:**
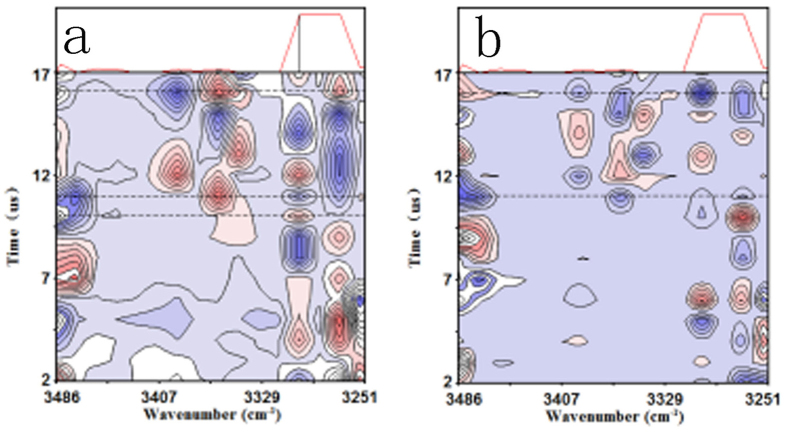
Perturbation correlation moving window 2D-COR FTIR contour maps of SPE-10 in the wavenumber range of 3250–3500 cm^−1^ under the external voltage of 4 V: (**a**) synchronous and (**b**) asynchronous spectra.

However, it is still difficult to obtain more details regarding the microcosmic dynamic transition behavior of the Li-ions under the EF by using such general 2D-COR FTIR technique. At the least, the following two questions are still needed to be further figured out. 1, whether is there a period during the real-time transition of the Li-ions under the EF. 2, if there is, how long is the period. Consequently, we have further utilized a novel perturbation-correlation moving window 2D-COR FTIR technique to real-timely track the dynamic migration behavior of Li-ions with the loading time scale of 1–20 μs and the corresponding results are shown in Fig. 4 for the SPE-10. As is shown in Fig. 4a, the intensity of characteristic peaks at around 3303 and 3358 cm^−1^ have been detected to alternately change with increasing the loading time from 1 to 20 μs. Especially, the intensity of the peak at around 3303 cm^−1^ significantly increases from zero to a maximum when the loading time ranges from 3.2 to 3.8 μs, then it gradually decreases to zero with further increasing the loading time to 5.0 μs. After that, the intensity of the characteristic peak maintains at around zero in the loading time scale of 5.0–7.0 μs. It further reduces from zero to a minimum when the loading time enhances from 7.0 to 8.2 μs, and gradually increases to zero when the loading time achieves to 9.5 μs. The intensity of the characteristic peak have been found to alternately increase (9.5–10.0 and 10.7–13.2 μs, positive) and decrease (10.2–10.7 and 13.5–16 μs, negative) with loading time, showing that Li-ions have periodically migrated along the direction of potential reduction by alternately dissociating and coordinating with the macromolecular ligands (lone pairs of the hydroxyl groups), and that the time scale for a critical migration of Li-ions in such PVA-LiClO_4_ electrolyte is actually at microsecond order of magnitude instead of other time scales (e.g. nanosecond, millisecond, even second). However, it can be observed that the time interval between different positive and negative peaks at about 3303 cm^−1^ is not maintained at a constant, which obviously implies that the variation of the macromolecular ligand under the EF (i.e. the corresponding microcosmic transition of Li-ions) with loading time is not a strictly periodic process but a quasi-periodic process. The quasi-period (*T*_*p*_) of Li-ions for the SPE-10 can be accordingly calculated from the interval between two neighboring positive or negative peaks, and the *T*_*p*_ value is found to range from 2.2 to 5.7 μs. In addition, the variation tendency of characteristic peaks in the synchronous contour map with the loading time can be extensively analyzed by tracking the intensity of corresponding peaks in the same bands (see Fig. 4b). Consequently, real-time dynamic transition information of Li-ions in the SPE-10 under the EF can be obtained by combining the value of characteristic peaks in the synchronous contour map with those in the asynchronous contour map. Detailed data are listed in Table 2. Similarly, microcosmic migration behavior of Li-ions for the SPE-8 and SPE-6 can be also detected using the same method (see Figure S4 and Table S3).

**Figure 5:**
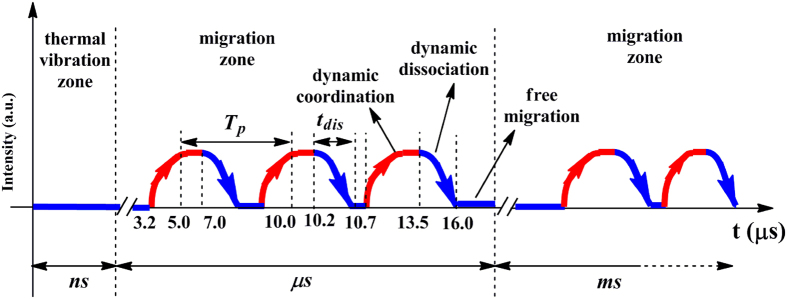
Schematically real-time transition behavior of Li-ions in the SPE-10 under the EF of 4 V.

For simplicity, the real-time dependence of the intensity of characteristic peak at about 3303 cm^−1^ for SPE-10 on the loading time is specially presented in Fig. 5. It is clearly shown that the intensity of the characteristic peak will alternately change when the loading time surpasses a certain critical value (c.a. 3.2 *μs*), actually implying the periodic transition of Li-ions under the EF. Meanwhile, the *T*_*p*_ values of 3.2 and 5.0 *μs* are simultaneously observed in the detected loading time scale, proving the above mentioned quasi-periodicity of the dynamic transition of Li-ions. The exact dynamic coordination number (*DCN*) for different SPEs under the EF can be obtained by dividing *T*_*p*_ values with corresponding *t*_*dis*_ values. The value of *T*_*p*_ and *t*_*dis*_ is apt to gradually enhance with Li-ion concentration, and the coordination form of SPE-10 and SPE-8 (two types) is more than that of SPE-6 (one type) (see Table S4). However, the *DCN* of the prepared SPEs is completely different from that of the original setting value (i.e. 10/1, 8/1, and 6/1), revealing the complicity of the dynamic transition of Li-ions under an EF. In addition, alternate variation of the intensity of corresponding characteristic peaks presented in Fig. 5 and Table 2 proves that the ionic transition under an external EF actually contains two relatively independent steps, namely, the dissociation (blue ramp sections of the curve in Fig. 5) of the coordinated ions and the diffusion (valleys of the curve) of the dissociated carriers. Apparently, the impediment during the transport of Li-ions derived from the interactions between Li-ion and hydroxyl groups bonded on the polymeric chains is closely related to the time interval of the increment, platform, and decrement of corresponding curves. Corresponding theoretical derivations with respect to dissociation and diffusion of Li-ions have been extensively discussed in our previous paper^21^,^22^. Finally, the maximum time internal of the peak platform gradually decreases from 2 to 1.4 and then to 0.8 *μs* when the original OH/Li ratio changes from 10/1 to 8/1 and then to 6/1.

## Conclusion

In summary, electrically induced real-time ionic migration behavior and transport dynamics in the PVA/LiClO_4_ electrolytes have been investigated by using a novel time-resolved FTIR combined with 2D-COR FTIR technique. The results for the first time confirm that there is no migration of Li-ions under an external EF when the time scale of the EF loading is at nanosecond (less than 200 *ns*). Li-ions have been found to significantly transfer along the direction of the EF when the time scale further enhances to microsecond orders of magnitude. The real-time migration of lithium cations in the SPEs under an EF is a complicated process accompanying with quasi-periodic dissociation and coordination effects between Li-ion carriers and polymeric chains. The quasi-period of Li-ions migration is detected to less than 10 *μs*. The time for Li-ions dissociated from polymeric chains has been monitored to be less than 3 *μs* by using perturbation correlation moving window 2D-COR FTIR technique. The exact *DCN* of the SPEs under the EF is completely different from that of the original setting value. The impediment during the transport of Li-ions originating from the interactions between Li-ion cations and hydroxyl group decreases with *DCN.* The developed combined method not only represents a new avenue in SPE research which is quantitative and powerful for the study of ionic transport dynamics, but also further enhances our understanding on the ionic conduction mechanism of SPEs.

## Additional Information

**How to cite this article**: Bao, L. *et al*. Real-time tracking the Li^+^-ion transition behavior and dynamics in solid Poly(vinyl alcohol)/LiClO_4_ electrolytes. *Sci. Rep.* **7**, 45921; doi: 10.1038/srep45921 (2017).

**Publisher's note:** Springer Nature remains neutral with regard to jurisdictional claims in published maps and institutional affiliations.

## Supplementary Material

Supplementary Information

## Figures and Tables

**Table 1:** Characteristic peaks of the SPE-10 with different loading time scale.

Characteristic peaks (cm^−1^)	*EF* = 0 *Vt* = 0–200 *ns*	*EF* = 4 *V*		
		*t* = 0–200 *ns*	*t* = 1~20 *μs*	*t* = 1–20 *ms*
3471	*D*	*D*	*D*	*D*
3448	*D*	*D→*	*N*	*D*
3400	*D*	*D*	*D*	*D*
3386	*D*	*D→*	*N*	*D*
3368	*D*	*D→*	*N*	*N*
3358	*N*	*N→*	*D*	*D*
3337	*D*	*D*	*D*	*D*
3303	*D*	*D*	*D*	*D*
3270	*D*	*D*	*D*	*D*

*D* and *N* mean that the characteristic peak is detected and not detected, respectively.

**Table 2:** Dynamic information of SPE-10 obtained from perturbation-correlation moving window 2D-COR FTIR spectra.

sample name	time scale (*μs*)	synchronous	asynchronous	variation type	quasi-period *T*_*p*_ (*μs*)	time of dissociation *t*_*dis*_ (*μs*)	coordination number (OH/Li)
SPE-10 (3303 cm^−1^)	3.2–3.8	+	0	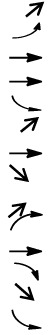	3.2, 5.0	0.5, 2.5	6.4/1, 2/1
	3.8–5.0	+	−				
	5.0–5.3	0	−				
	5.3–7.0	0	+				
	7.0–9.5	−	0				
	9.5–10.0	+	0				
	10.0–10.2	0	0				
	10.2–10.7	−	0				
	10.7–12.0	+	0				
	12.0–13.2	+	+				
	13.2–13.5	0	+				
	13.5–13.9	−	+				
	13.9–15.4	−	+				
	15.4–16.0	−	−				
