# Supplementary material for: Real-time tracking the Li+-ion transition behavior and dynamics in solid Poly(vinyl alcohol)/LiClO4 electrolytes
Source: Sci Rep. 2017 Apr 5;7:45921. doi: 10.1038/srep45921 (PMC5381101; doi:10.1038/srep45921)
Supplement: Supplementary Information [file srep45921-s1.pdf]

Real-time tracking the Li^+-ion transition behavior and dynamics in solid Poly(vinyl alcohol)/$LiClO_4$ electrolytes

Lixia Bao[†], *Xin Zou*[†], *Xin Luo*[†], *Yanlei Pu*[†], *Jiliang Wang*[*†], *Jingxin Lei*[*‡]

†. School of Chemical Science and Technology, Yunnan University, Kunming 650091, P.R. China.

‡. State Key Laboratory of Polymer Materials Engineering, Polymer Research Institute of Sichuan University, Chengdu 610065, P.R. China.

Corresponding Author

*Email: jlwang@ynu.edu.cn

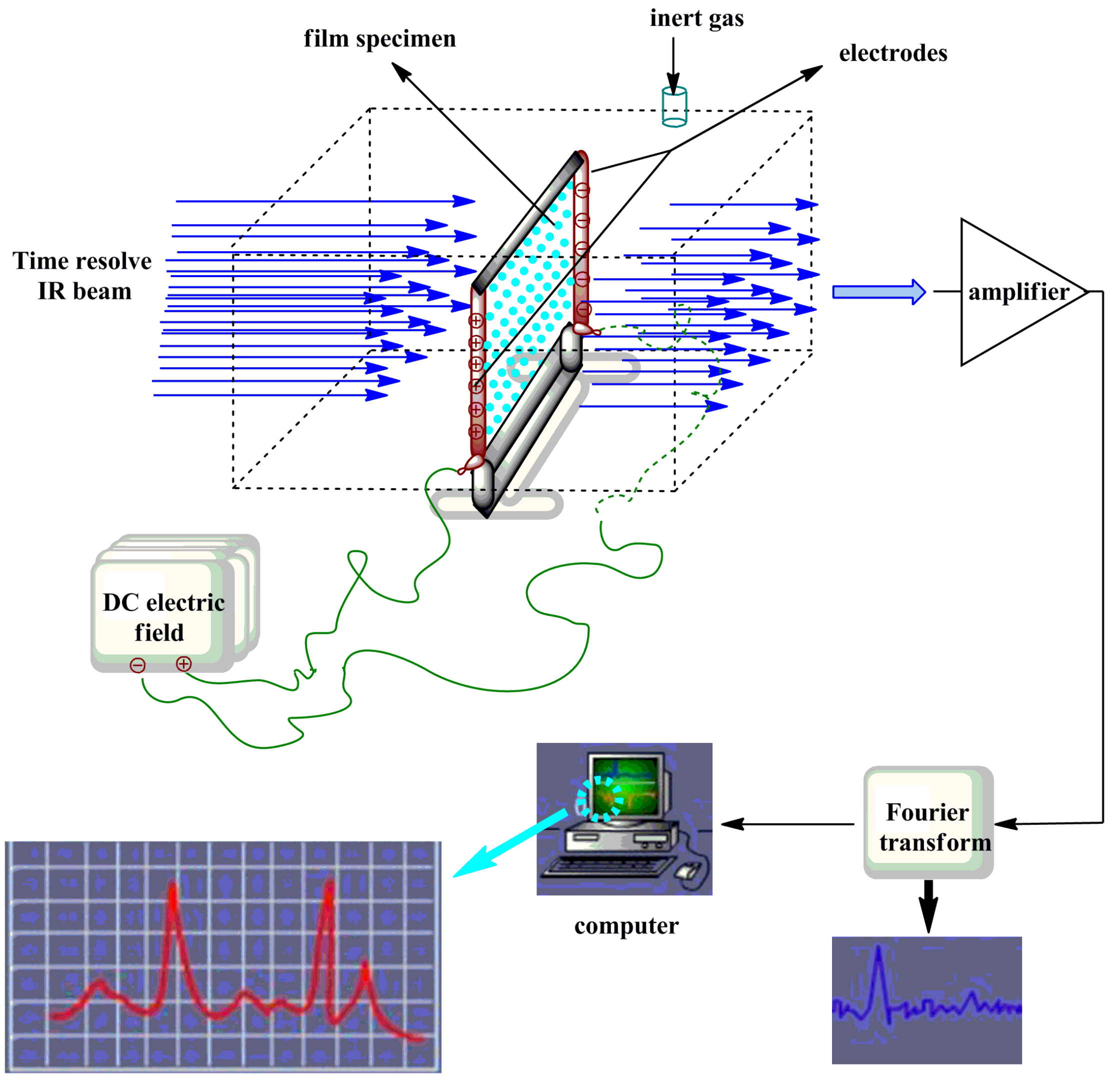

Figure S1. Schematic test route of the TR-FTIR measurement

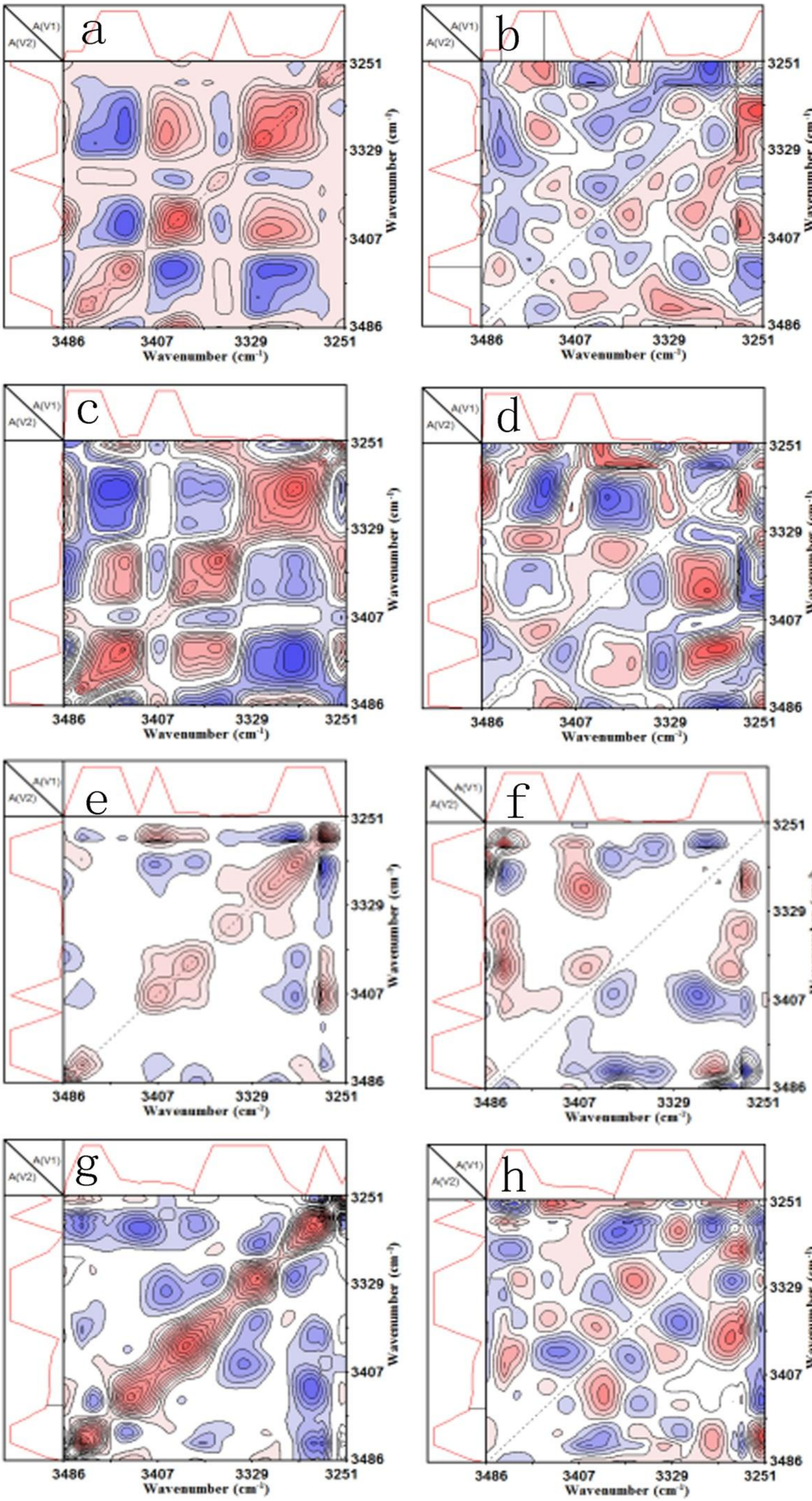
a
b
c
d
e
f
g
h
A(V1)
A(V2)
3486
3407
3329
3251
Wavenumber (cm⁻¹)

Figure S2. 2D-COR FTIR contour maps of the SPE-8 in the wavenumber range of 3250-3500 cm^{-1} without EF (a, b) and with the external voltage of 4 V (c-h), (a,c,e,g) synchronous spectra and (b,d,f,h) asynchronous spectra.

Table S1. Characteristic peaks of the SPE-8 with different loading time scale

Characteristic peaks (cm^{-1})	*EF* = 0 *V*	*EF* = 4 *V*		
	t = 0-200 *ns*	t = 0-200 *ns*	t = 1~20 μs	t = 1-20 *ms*
3471	D	D	D	D
3440	D	D	D	D
3400	N	N→	D	D
3386	D	D	D	D
3368	D	D→	N	N
3358	N	N→	D	D
3337	D	D	D	D
3303	D	D	D	D
3270	D	D→	N	D

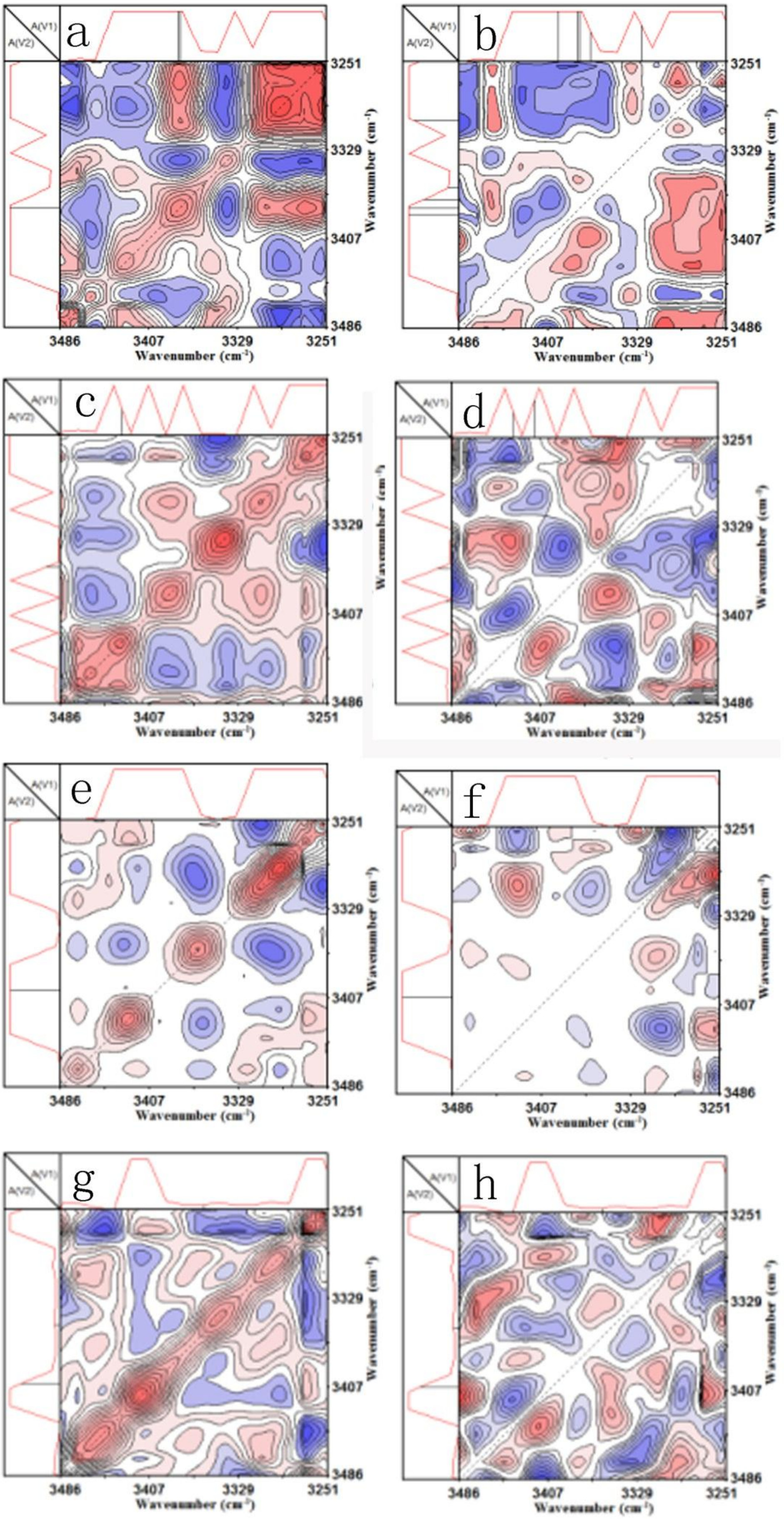
a
b
c
d
e
f
g
h
A(V1)
A(V2)
3486
3407
3329
3251
Wavenumber (cm-1)

Figure S3. 2D-COR FTIR contour maps of SPE-6 in the wavenumber range of 3250-3500 cm^{-1} without EF (a, b) and with the external voltage of 4 V (c-h), (a,c,e,g) synchronous spectra and (b,d,f,h) asynchronous spectra.

Table S2. Characteristic peaks of the SPE-6 with different loading time scale

Characteristic peaks (cm^{-1})	$EF = 0\ V$	$EF = 4\ V$		
	t = 0-200 ns	t = 0-200 ns	t = 1-20 μs	t = 1-20 ms
3471	D	D	D	D
3440	D	D	D	D
3400	D	D→	N	D
3386	D	D→	N	D
3368	D	D→	N	N
3358	N	N→	D	D
3337	N	N	N	D
3329	D	D	D	N
3303	N	N→	D	D
3270	D	D	D	D

It is obviously presented in Table S1 and S2 that characteristic peaks are also almost unchanged at the loading time scale of 0-200 *ns*, even though the external EF has been changed from 0 to 4 V. However, at least four characteristic peaks are found to be significantly changed once the loading time increases to microsecond order of magnitude (1-20 μs), showing a similar variation tendency in comparison with that of the SPE-10 (see Table 1). In addition, the variation sequence of -OH groups under the EF with the loading time scale of 1-20 μs for the SPE-8 and SPE-6 can be accordingly obtained as follows.

$$\text{(SPE-8) } 3337 \rightarrow 3386 \rightarrow 3303 \rightarrow 3358 \rightarrow 3400 \rightarrow 3440 \rightarrow 3471\ cm^{-1}$$

$$\text{(SPE-6) } 3471 \rightarrow 3440 \rightarrow 3358 \rightarrow 3329 \rightarrow 3303 \rightarrow 3270\ cm^{-1}$$

Consequently, microcosmic dynamic transition behavior of Li-ions in the SPE-8 and SPE-6 under the external EF can be delicately detected by using similar method as well (similar with Figure 3).

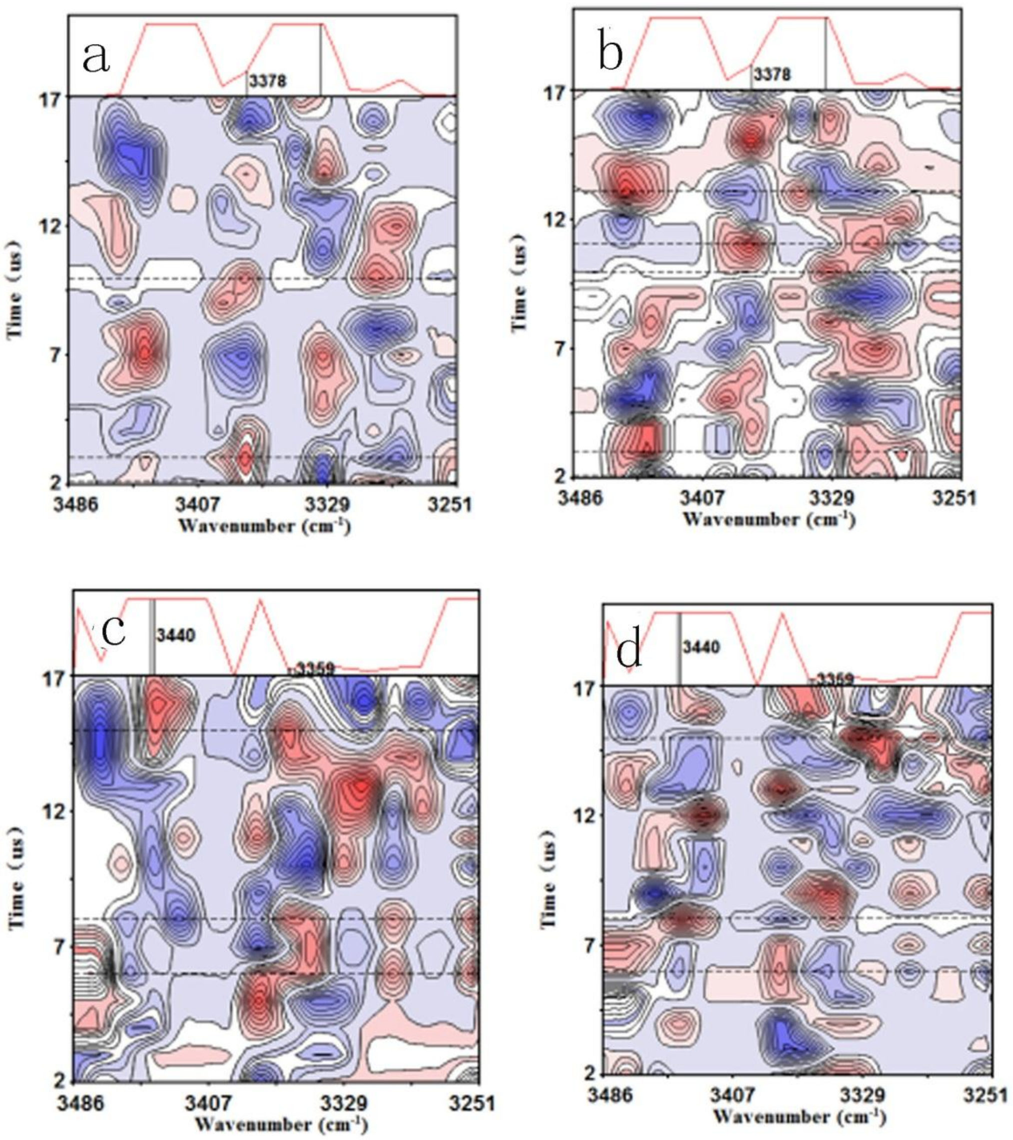

Figure S4. Perturbation correlation moving window 2D-COR FTIR contour maps of SPE-8 and SPE-6 in the wavenumber range of 3250-3500 cm^{-1} under the external voltage of 4 V: (a,c) synchronous spectra of SPE-8 and SPE-6, respectively, and (b,d) asynchronous spectra of SPE-8 and SPE-6, respectively.

Table S3 Dynamic information of SPE-8 and SPE-6 obtained from perturbation-correlation moving window 2D-COR FTIR spectra

sample name	time scale (μs)	synchro-nous	Asynchro-nous	variation type	quasi-period T_p (μs)	time of dissociation t_{dis} (μs)	Coordination number (OH/Li)
SPE-8 (3378 cm^{-1})	2-4.3	+	0	↗	4.4, 6.4	0.8, 2.7	5.5/1, 2.4/1
	4.3-5.3	0	+	→			
	5.3-7.0	-	+	⤵			
	7.0-7.5	-	0	↘			
	7.5-8.0	-	-	⤷			
	8.0-8.5	0	-	→			
	8.5-9.5	+	-	⤴			
	9.5-9.8	+	0	↗			
	9.8-10.5	+	+	↱			
	10.5-11.7	0	+	→			
	11.7-11.9	0	0	→			
	11.9-12.5	-	-	⤷			
	12.5-13.0	0	-	→			
	13.0-14.3	+	-	⤴			
	14.3-14.5	+	0	↗			
	14.5-15.1	0	+	→			
	15.1-16.2	-	+	⤵			
	16.2-16.4	-	0	↘			
	16.4-17.0	-	-	⤷			
SPE-6 (3378 cm^{-1})	3.3-4.5	+	-	⤴	7.1	3.2	2.2/1
	4.5-4.7	+	0	↗			
	4.7-5.9	+	+	↱			
	5.9-6.3	0	+	→			
	6.3-7.2	-	+	⤵			
	7.2-7.4	-	0	↘			
	7.4-8.2	-	-	⤷			
	8.2-9.5	-	0	↘			
	9.5-10.3	0	-	→			
	10.3-10.9	+	0	↗			
	10.9-12.0	+	-	⤴			
	12.0-12.1	+	0	↗			
	12.1-13.0	+	+	↱			
	13.0-13.3	0	+	→			
	13.3-13.4	-	0	↘			
	13.4-15.2	-	-	⤷			
	15.2-17.0	-	0	↘			
